# HCV resistance

**DOI:** 10.1186/1742-4690-9-S1-I18

**Published:** 2012-05-25

**Authors:** Philippe Halfon

**Affiliations:** 1 Ambroise Paré Hospital, Marseille, France

## 

The efficacy of Direct Antiviral Agents (DAAs) is limited by the presence of Resistant associated virus mutations (RAVs) resulting in amino-acid substitutions within the targeted proteins which affect viral sensitivity to these compounds. Six major position mutations in the NS3 HCV Protease (36, 54, 155, 156, 168, and 170), fifteen in the NS5B polymerase (96, 282, 316, 365, 414, 419, 423, 448, 482, 494, 495, 496, 499, 554, 559) and five in the NS5 A region (28, 30, 31, 58 and 93) have now been reported *in vitro* or *in vivo* associated with different levels of resistance.

The HCV NS3,NS5A, Pol (NNI) mutations occurred quickly (less than 15 days) and longer for Nucleosides Inhibitors in monotherapy and the genetic barrier can be overcome by combination with Pegylated-Interferon+Ribavirine in quadruple therapy. There is a long term persistence of HCV NS3 Protease mutations after the end of therapy and it is important to stop the NS3 protease inhibitor early in patients with ongoing replication to avoid the selection of resistant variants with increased fitness and a higher potential of long-term survival. Issues on HCV archived mutations are not solved. Virological failure was more likely in patients with genotype 1a infection than 1b and was associated with the presence of resistant variants. Furthermore, the time taken for resistant HCV variant populations to return to WT is longer for patients with genotype 1a versus genotype 1b.

Selection of resistant variants that, in turn, could produce cross-resistance to whole class of drugs with overlapping resistance profiles Combinations of Protease Inhibitors with other class of antiviral with separate modes of action & non-overlapping resistance profile is preferable. Additionally, Ribavirine prevents viral breakthrough in combination with Pegylated-Interferon and DAAs and the effect of ribavirin important seems limit the DAA resistance.

The resistance profiling does remain a challenge for the next generation of protease, NS5A, non nucleoside inhibitors and probably for nucleoside inhibitors; thus, the lessons from HIV infection and the first clinical proof of IFN-free regimen treatment indicate that combinations of drugs with different mechanisms of action will be an attractive strategy for hepatitis C.

